# The Integration of Psychosocial Care into National Dementia Strategies across Europe: Evidence from the Skills in DEmentia Care (SiDECar) Project

**DOI:** 10.3390/ijerph18073422

**Published:** 2021-03-25

**Authors:** Ilaria Chirico, Rabih Chattat, Vladimíra Dostálová, Pavla Povolná, Iva Holmerová, Marjolein E. de Vugt, Niels Janssen, Fania Dassen, María Cruz Sánchez-Gómez, Francisco José García-Peñalvo, Manuel A. Franco-Martín, Giovanni Ottoboni

**Affiliations:** 1 Department of Psychology, University of Bologna, 40126 Bologna, Italy; rabih.chattat@unibo.it (R.C.); giovanni.ottoboni@unibo.it (G.O.); 2 Faculty of Humanities, Charles University, 182 00 Prague, Czech Republic; Vladka.Dostalova@seznam.cz (V.D.); povolna@ipvz.cz (P.P.); iva.holmerova@gerontocentrum.cz (I.H.); 3 Institute for Postgraduate Medical Education, Charles University, 100 00 Prague, Czech Republic; 4 Alzheimer Centrum Limburg, Maastricht University, 6229 MD Maastricht, The Netherlands; m.devugt@maastrichtuniversity.nl (M.E.d.V.); niels.janssen@maastrichtuniversity.nl (N.J.); f.dassen@maastrichtuniversity.nl (F.D.); 5 Psycho-Sciences Research Group of IBSAL, Salamanca University, 37007 Salamanca, Spain; mcsago@usal.es (M.C.S.-G.); fgarcia@usal.es (F.J.G.-P.); mfm@intras.es (M.A.F.-M.)

**Keywords:** psychosocial care, psychosocial intervention, policy, national strategies, dementia, Europe, health priorities, quality of life, Alzheimer’s disease, caregivers

## Abstract

There is evidence supporting the use of psychosocial interventions in dementia care. Due to the role of policy in clinical practice, the present study investigates whether and how the issue of psychosocial care and interventions has been addressed in the national dementia plans and strategies across Europe. A total of 26 national documents were found. They were analyzed by content analysis to identify the main pillars associated with the topic of psychosocial care and interventions. Specifically, three categories emerged: (1) Treatment, (2) Education, and (3) Research. The first one was further divided into three subcategories: (1) Person-centred conceptual framework, (2) Psychosocial interventions, and (3) Health and social services networks. Overall, the topic of psychosocial care and interventions has been addressed in all the country policies. However, the amount of information provided differs across the documents, with only the category of ‘Treatment’ covering all of them. Furthermore, on the basis of the existing policies, how the provision of psychosocial care and interventions would be enabled, and how it would be assessed are not fully apparent yet. Findings highlight the importance of policies based on a comprehensive and well-integrated system of care, where the issue of psychosocial care and interventions is fully embedded.

## 1. Introduction

Dementia is one of the major global causes of disability and dependency among older people [1]. Nowadays, it is considered a global public health priority [2]. In Europe, 10 million people have been diagnosed with dementia. The majority of them are cared for by informal carers, i.e., relatives and/or friends who may be exposed to physical, emotional and economic burdens causing severe consequences for own their health [3]. High-quality and multidisciplinary interventions are urgently needed to help people with dementia and their carers to cope with symptoms and to improve their quality of life. Among them, there are a growing number of psychosocial interventions with established effectiveness [4,5,6,7,8].

Psychosocial interventions encompass physical, cognitive or social activities (Table 1). They have a twofold aim: on the one hand, they attempt to maintain or improve personal functioning, interpersonal relationships and well-being in people with dementia; on the other hand, they try to minimize the risk of future disability [5,9]. During the design and the development of these interventions, person’s history, family and social context, and stigma reduction are taken into account [10].

Empirical evidence has shown that psychosocial interventions are more cost-effective and have less side effects than anti-dementia drugs [8]. Moreover, there is evidence for their effectiveness across several areas of individual functioning, and different types and stages of dementia [7]. A recent synthesis of systematic reviews [5] found that a multi-component exercise with sufficient intensity improves people with dementia’s physical and cognitive functions, as well as their daily living activities. Moreover, the use of group cognitive stimulation was associated with people with dementia’s increased cognitive abilities, social functioning and quality of life.

Nowadays, psychosocial interventions are highly recommended for treating people with dementia’s neuropsychiatric symptoms and behavioural changes that are particularly difficult to manage by family and/or friends [11,12,13]. Environmental adjustments, such as lifestyle support, are generally first-line interventions [14]. For example, in this context, individualized music therapy, bright light treatment, and aromatherapy have been found to positively impact people with dementia’s agitation and aggression [15,16]. Furthermore, teaching caregivers techniques to manage people with dementia’s behaviour problems can make home living less challenging for both the person with dementia and family members [17]. Specifically, there is good evidence that group interventions consisting of both educational components focused on enhancing caregivers’ knowledge of dementia and the caring role, and having a therapeutic component (for example, cognitive behavioural therapy), reduce caregivers’ psychological burden and delay institutionalization [4,18,19,20,21,22,23]. Moreover, incorporating a technological component via telephone/online support could be more cost-effective [4].

However, despite the evidence, concrete actions to translate research into practice are sparse and inconsistent worldwide [2]. A lack of public and specific specialist/professional knowledge about dementia often results in stigmatization and barriers to diagnosis and appropriate care. Hence, dementia is often under and/or not timely diagnosed, leading to fragmented or completely lacking care pathways [1]. Moreover, investments in drug discovery and development have become mostly unsuccessful [24], making it urgent to establish which treatments are effective for people with dementia and their carers.

In this context, policies play a crucial role in addressing dementia challenges and establishing what is needed to meet these challenges to improve care quality. A recent review [25] analyzed global and European dementia policies to identify current challenges and gaps. Results depicted a scenario of high inconsistency and fragmentation with dramatic consequences which could affect, in particular, low and middle-income countries in the next years. Indeed, a significant variability was found among the policy documents, ranging from drafts to full strategies and plans whose implementation is mostly beginning with an unknown impact. The authors concluded that each country should set its national priorities, i.e., what it is needed to achieve for its citizens with dementia and their carers.

To our knowledge, no previous studies have been conducted investigating whether and how the issue of psychosocial care and interventions has been addressed in the national dementia plans and strategies across Europe. Due to the importance of implementing psychosocial interventions in everyday practice, the present study aims to analyze European policy documents and check whether they include reference, or full sections, devoted to psychosocial care and interventions.

This study was one of the two pillars of the Erasmus+ project entitled ‘Skills in DEmentia Care-Building psychosocial knowledge and best practice in dementia care’ (SiDECar) [26]. The two pillars form the basis for developing higher education curricula of studies to deliver an evidence-based, and well-systematized body of knowledge and skills on psychosocial care in dementia [27].

## 2. Materials and Methods

The search and analysis of European dementia plans and strategies lasted from January 2019 to October 2020. Specifically, the search interested only those countries whose organizations are members of the Alzheimer Europe (Table 2).

At first, documents were searched on the Alzheimer Europe website [28] and, if they were not indicated in that website, Alzheimer Disease International website was investigated [29]. If no document was available from any of the two websites, the search was performed using the Google search engine.

Since the Cypriot dementia strategy was advertised on the Alzheimer Europe website, although not retrievable, the authors sent a support request to AE to include this document in the list. Belgium provided the strategy of one region only, i.e., Flanders. For what concerns the United Kingdom (UK), the strategies were provided per countries, i.e., England, Northern Ireland, Scotland, and Wales. Once the documents were retrieved, if needed, they were fully translated into English by two authors (IC, VD).

Initially, all the documents were thoroughly read to check whether they include references to psychosocial care and interventions. Subsequently, the parts of the texts focused on psychosocial care and interventions were marked and analyzed by content analysis [30,31]. Thus, the relevant parts of the texts were summarized in codes, and grouped into categories and subcategories according to similarity across the codes [32]. Data analysis was performed independently by two researchers experienced in content analysis (VD, PP). Disagreements were resolved through discussion with a third author (IC). All co-authors approved every step of this analysis. The coding structure with categories, subcategories and codes is included in Supplementary Table S1.

## 3. Results

As shown in Table 2, a total of 26 national documents were found: 15 derived from AE, eight from ADI, 2 from the Google search, and one (i.e., the Cypriot strategy) was available on request. All documents were published between the years 2009 and 2020.

Five documents were available in English as coming from English-speaking countries (Ireland, England, North Ireland, Scotland, Wales). The ones translated into English by the national governments were eight (Belgium, Denmark, Finland, Greece, Israel, Malta, Norway, Switzerland). Two documents (Italy, Luxembourg) were available in an unofficial English translation. The remaining documents (Austria, Cyprus, Czech Republic, France, Germany, Iceland, Netherlands, Portugal, Slovenia, Spain, Sweden) were translated into English.

The content analysis revealed that the main categories associated with psychosocial care and interventions were homogeneous across the documents. Specifically, three main pillars emerged: (1) Treatment, (2) Education, and (3) Research.

Moreover, the analysis suggested dividing the first category into three subcategories: (1) Person-centred conceptual framework, (2) Psychosocial interventions, and (3) Health and social services networks.

Overall, as shown in Table 2, the topic of psychosocial care and interventions was addressed in all the 26 national documents, with the category of ‘Treatment’ covering all of them. However, while the discussion was quite general in some documents, in others, more detailed information was provided.

### 3.1. Treatment

Results show an emphasis on the increasing referral to psychosocial care for people with dementia as an integral part of dementia care in all the reviewed documents. The focus is intended in terms of a multidisciplinary approach involving medical treatments and psychosocial interventions to improve people with dementia’s symptomatic profile, quality of life, and social inclusion. Specifically, regarding the prevention and management of the behavioural and psychological symptoms of dementia (BPSD), the Irish National Plan explicitly states that antipsychotic drugs should be used only when psychosocial interventions are not effective. Similarly, in the Swedish National Plan, psychosocial care is highly recommended as the first-line approach in dealing with BPSD, and consists of an adaptation of physical and social environment.

#### 3.1.1. Person-Centred Conceptual Framework

Concerning the conceptual framework underpinning the interventions for people with dementia, reference is made to the nature of the psychosocial concept of care. As reported by the Austrian National Dementia Strategy, this perspective emphasizes the importance of the individual-centred needs assessment, focusing on people with dementia’s personal abilities and resources. Similarly, the Northern Ireland Dementia Strategy explicitly states that psychosocial interventions should promote people with dementia’s independence, while maintaining a good functioning in terms of physical and cognitive skills, emotional and psychological well-being. The Portuguese Dementia Strategy recommends a person-centred care model focusing on including people with dementia and their families in society, while encouraging their active participation in community life. The Maltese Strategy mentions the importance of developing programs of purposeful and therapeutic activities that maintain the person with dementia active and engaged in meaningful occupations. Similarly, the Swedish National Dementia Strategy recommends implementing different support measures to cover several needs of people with dementia, including special housing and day-to-day activities. Furthermore, an innovative element of the Dutch National Plan concerns the development of e-health applications, including domotics, to foster people with dementia’s autonomy, and shared decisions through different disease stages.

#### 3.1.2. Psychosocial Interventions

Concerning clinical practice, reference is made to specific types of psychosocial interventions, such as physical activities (Cyprus, Denmark, France, Germany, Greece, Malta, Norway, Portugal, Spain, Sweden, England, Wales), cognitive stimulation (Cyprus, France, Greece, Norway, Portugal, Spain, Wales), music therapy (Norway, Portugal, Sweeden, England), and occupational therapy (Cyprus, Greece, Portugal, Wales). Speech therapy (Cyprus, Greece), sensory stimulation (Cyprus, Portugal), and art therapy (Greece, England) are also recommended as appropriate psychosocial interventions. The Portuguese Dementia Strategy recommends the use of garden walks, cooking, animal-assisted therapy, Snoezelen, reminiscence, play activities, multisensory and motor stimulation, and hydrotherapy. Several strategies (Belgium, Denmark, England, Germany, Ireland, Malta, Northern Ireland, Norway, Portugal, Slovenia, Wales) emphasize the need to ensure dementia-sensitive environments, which can improve people with dementia’s sense of direction, mobility, and safety within residential care settings, hospitals, community health services, private home, shops or public spaces. New buildings should be constructed or renovated to become friendly, inclusive, and supportive for people with dementia. For example, according to the Irish National Plan, hospital wards should be carefully designed in terms of environment, i.e., safe walking spaces, use of colour, lighting, signage, orientation cues and space used to promote social interactions. The Norwegian National Strategy recommends constructions to be based on universal design principles, adapted to people with dementia and their impairments, and equipped for the use of electronic aids to daily living, such as alarm technology, and other welfare devices.

#### 3.1.3. Health and Social Services Networks

Cooperation and networking among health and social services are also mentioned in all the documents. In particular, the Greek Action Plan for Dementia and Alzheimer’s Disease stresses the importance to strengthen the existing day-care centres of the Psychargos program [34] with multi-professional teams (e.g., nurses, occupational therapists, social workers). The Pillar number 2 in the Dutch Dementia Plan, called ‘Dementia care for each other’, aims to provide customized support to partnerships through a knowledge network where available knowledge, good practice, experiences, and tools are easily accessible digitally or live, via thematic meetings. Furthermore, the Objective number 6 in the National Dementia Strategy of England highlights the need to integrate community services to obtain a more straightforward route to access services, and a more coordinated delivery of services.

### 3.2. Education

The need for appropriate education and training about psychosocial interventions features 12 documents (Czech Republic, Denmark, Finland, France, Ireland, Italy, Malta, Netherlands, Norway, Portugal, Slovenia, Spain). Specifically, nine of them highlight the importance to develop and offer adequate programs of education and trainings both for professional carers from different specialities (Denmark, Finland, France, Malta, the Netherlands, Norway, Portugal, Slovenia, Spain), and informal carers (Ireland, Portugal, Spain).

A common principle is that education should be evidence-based and provide professionals with knowledge and skills on how to deliver psychosocial interventions in different care settings. Moreover, professionals should be trained to support people with dementia’s identity and quality of life until the later stages of the disease.

Both the French and Spanish National Plans refer to cognitive stimulation as a content upon which education should focus. Furthermore, the Italian National Dementia Strategy stresses the need to develop documents and guidelines based upon experts’ consensus, and to guarantee continuing education on psychosocial interventions. At the same time, this document does not specify any education target group.

Similarly, the need to develop and disseminate handbooks with related knowledge-based recommendations are mentioned in the Czech Republic’s, Danish and Dutch National Dementia Plans. Such proposals aim to strengthen social and health professional practice and ensure a shared and coherent approach in dementia. Specifically, the Dutch Dementia Plan includes a training program called ‘Dealing with dementia’ aimed at a dementia-friendly society. It offers specific evidence-based courses for different target groups, i.e., individuals, companies and municipalities, to increase public awareness about dementia, and people’s ability to cope with this chronic condition.

The Portuguese Dementia Strategy mentions the importance of providing education on health, psychosocial and related fields for students (during undergraduate and postgraduate studies), formal and informal caregivers. According to the Norwegian Dementia Plan, the ‘Dementia ABC education program’ and the ‘Psychosocial Intervention ABC educational program’ should be developed and promoted by the Ministry of Health and Care Services for municipal health and care services personnel.

### 3.3. Research

References to research are included in seven out of 26 documents (Finland, France, Germany, Ireland, Malta, Netherlands, Spain). Emphasis is placed on the need to translate the knowledge already gained in this area into practice, and increase the quality of care for people with dementia via data collecting policies about the effectiveness of psychosocial interventions. Finland’s National Memory Program states that more studies are needed to better understand the implementation of psychosocial interventions into routine clinical practice, including technological innovations, and to disseminate these results across services. The German National Dementia Strategy suggests that ecological studies may investigate the weight of the various psychosocial factors, along with research on the optimization of healthcare processes. Furthermore, the Spanish National Dementia Plan emphasizes the need to support research on the physical and psychosocial needs of people with dementia, as to develop innovative models of care or technologies. Similarly, the Dutch Dementia Plan mentions the importance of research on innovations and new technology, including home automation.

## 4. Discussion

This study investigates whether and how the issue of psychosocial care and interventions is dealt within the national dementia strategies and plans across Europe. Results have shown that, at different levels of detail, all the documents refer to a model of integrated care and support consisting of medical treatments and psychosocial interventions. The analysis has identified 3 main pillars of psychosocial care and interventions: ‘Treatment’, ‘Education’, and ‘Research’.

Regarding the category ‘Treatment’, it is generally recognized that psychosocial interventions should enable people with dementia to retain their functional ability and autonomy, reduce behavioural and psychological symptoms, and improve their quality of life. The theoretical framework represented by the *person-centred* model of care [35,36] implies that people with dementia should be valued as persons with the same dignity as others, and should be treated with respect as for their own life history, experiences, personality, as well as for the cultural and social contexts to which they belong. This idea is central as it is strictly associated with the formulation, choice and use of psychosocial interventions. A similar approach is radically different from the ‘industrialized’ vision of the care where people are dealt with as a series of tasks by professionals [7].

The *person-centred* (preferable to the alternative *patient-centred*) model of care highlights the importance of taking actions tailored to the individual needs, desires and preferences, which are immanent in people well beyond their disease. In this context, attention must be paid to people with dementia’s active participation and inclusion in the community life.

Concerning clinical practice, more than half of the documents mention various psychosocial interventions including physical activities, cognitive stimulation, music, occupational, speech and art therapy, sensory stimulation and so on. In this context, the focus is also on the environmental design, which needs to be carefully considered to help people with dementia to find their way around, reduce confusion and increase independence. The underpinning idea is that people are as constrained as the environment causes them to be. In this sense, attention should be paid to the use of natural light, colour coding for walls, personalized doors, orientation cues, gardens with different areas to encourage different sensory experiences and so on.

Although some information is provided within the documents, the targets of psychosocial interventions for people with dementia are often omitted (e.g., functional abilities, behaviour, emotions). No specifications are also provided for their use in different types and stages of dementia. Furthermore, since the empirical support for various types of psychosocial interventions differs [5], this aspect should be addressed by existing policies to provide appropriate care.

Moreover, it is important to underline that the area of psychosocial interventions for informal carers is widely neglected. Indeed, while a general need for support has been mentioned, no indication of specific interventions has been provided [4].

The analyzed documents also highlight that professionals from different services should collaborate when handling matters concerning people with dementia and their families to provide holistic and integrated health and social care. This aspect is crucial since the complex range of cognitive, physical, social, and emotional issues that dementia rises cannot be easily managed by a single professional. In this regard, positive effects of using a multidisciplinary diagnostic approach have been found on people with dementia’s health-related quality of life and confirmed at follow-up [37]. For example, in the Netherlands, by stimulating collaboration, *DementiaNet* has enhanced professional knowledge and skills and increased quality of care and clinicians’ ability to take leadership roles in a collaborative network [38]. In Germany, dementia care networks include a growing number of community-based support services for people with dementia and their caregivers. They offer personal care and support while providing a single entry point to social services, thus overcoming the interface problem [39].

Concerning the second pillar ‘Education’, the emphasis is placed on the importance of education and training programs. However, targets (i.e., professions, staff qualifications, informal carers), features, and potential courses’ contents are often unmentioned. In the case they are conceived for professionals, the education and training for families would remain strongly neglected with severe consequences for the considerable amount of informal and unpaid caregivers in need of support and care [27]. It seems that, nowadays, the quality of care mostly depends on each professional’s theoretical and practical knowledge, personal and professional experiences and, not least, on the rules of the institutions/services/agencies he/she works for. Consequently, much more effort should be deployed to establish the best practices for social and health care practitioners. The final aim is to ensure adequate support and evidence-based interventions for people with dementia and their families.

Concerning the last category ‘Research’, although it results in a few documents only, it underlines the need to carry out studies on the effectiveness of psychosocial interventions to understand better how interventions work in practice. Indeed, as highlighted in the literature [7,10,40], many complex interventions are not evaluated to a standard for different reasons. Among them are a lack of standardized measurement instruments for process evaluations, and the fact that these assessments may be time-consuming, and of less interest than effect analyses.

Overall, the amount of information provided differs across the country policies, with only the category of ‘Treatment’ covering all of them. Furthermore, on the basis of these documents, how the provision of psychosocial care and interventions would be enabled, and how it would be assessed are not fully apparent yet.

Such a scenario could reflect recent research findings suggesting that, although progress has been made in developing and evaluating psychosocial interventions, just few of them are widely accepted and implemented among different regions [22,40]. Reasons are several. Firstly, many interventions neither have a practice manual nor a specific description of the process, making it difficult to replicate in practice. Moreover, very few data are available on the acceptability of the interventions to the target/s, which may directly impact their dissemination and use [22,41,42]. Specifically, the acceptability refers to intended recipients’ judgment on whether intervention procedures are appropriate, fair, and reasonable [43]. A Japanese study [44] on the DEMBASE^®^ program has found that the facilitators for implementing psychosocial interventions include program available for care managers and offered at no charge, feedback on professionals’ work, and media coverage (e.g., nationwide newspapers and television). The barriers include professionals from different organizations who find it challenging to participate in interdisciplinary discussion meetings; and unpaid work as there is often no compensation for additional time associated with training and supervision. Results from a Dutch study [45] on the implementation of the Dementia Care Mapping in care homes show that facilitators are: professional’s confidence, ability to engage staff members, and effective leadership within the organization. Instead, challenges are: high staff turnover rates, low staff educational levels and confidence, lack of time, and managerial or organizational support. Similarly, Kloos and colleagues [46] have found a range of determinants including teamwork, leadership, and organizational factors such as staffing, workload, flexibility of the organization, and availability of a clear implementation plan.

Although research in this area is still limited, it is important to highlight that barriers and facilitators are country-specific (i.e., depending on culture, socio-economic factors). Such an element should be part of the following analysis about the impact of barriers and facilitators on intervention plans. To this aim, since the culture of the care home and systems issues are crucial, ‘bottom-up’ approaches should involve home staff, managers and providers in the design of interventions [45].

### Strengths and Limitations

Strengths include the comprehensive overview of national dementia strategies and plans of European countries. To our knowledge, no studies so far have focused on the issue of psychosocial care and interventions in the context of national dementia strategies and plans across Europe. In doing so, this study has been carefully planned, and a priori and well-defined qualitative research methodology has been used by starting from a systematic search strategy of all documents. Every stage of this project has been accurately evaluated and monitored by a panel of researchers. Nevertheless, some limitations should be kept in mind when reading our results. Local policies are not the focus of our study and, therefore, have not been included. Another question is also to what extent these policy documents reflect the actual clinical practice in each country. As previously discussed, research in this area is very recent, and future efforts should be put into the identification and understanding of the barriers to the implementation of psychosocial interventions into practice and, consequently, how to deal with them [47].

## 5. Conclusions

Key points and recommendations are reported in Table 3. if a well-defined set of policies and procedures are needed to regulate health and social systems, no strategy, plan or policy will be successful without proper political efforts, adequate financial investments, service accessibility, and appropriate organizational structures. Multiple and simultaneous efforts by different stakeholders are required, and all of them should be based on a shared vision, values and practices when working in this field. Policies should be harmonized across Europe and based on a comprehensive and well-integrated system of care, where psychosocial care and interventions are fully developed. Specifically, each document should provide a clearer picture of how psychosocial care and interventions would be enabled. Research is necessary on the country-specific basis to investigate the impact of barriers and facilitators upon implementing the psychosocial interventions. Well-designed education and training programs are needed for students and both formal and informal caregivers. Finally, as working groups of people with dementia, public and private associations of formal and informal carers are growing worldwide, policy should promote and facilitate public awareness about dementia, and the development of more dementia-friendly societies. All these actions, taken together, can contribute to the improvement of dementia care, policies, and psychosocial professional culture.

## Figures and Tables

**Table 1:** List of psychosocial interventions.

Interventions	Definition/Examples
Carer interventions	Psychoeducation, cognitive behavioral therapy, counselling
Physical activities	Seated exercise, walking, strength training
Reminiscence	Therapy based on the use of human senses to help people with dementia remember events, people and places from their past
Multisensory stimulation/Snoezelen	Non-directive therapy aimed at providing a multi-sensory experience or single sensory focus, by adapting the lighting, atmosphere, sounds, and textures to the person’s needs
Massage/touch	Regular massage forms (i.e., a touch with some pressure is applied in a moving way on parts of the body); therapies focused on finger pressure on specific points; ‘therapeutic touch’ (i.e., interventions where the therapist’s hands may be held at a short distance from the person’s body)
Behavior management	Techniques based on the ABC model where the focus is on identifying the A (antecedent or activating event), that lead to the B (challenging behavior), and examining the C (consequence) of the behavior. The aim is to improve carer’s ability to identify and reduce triggers for behavioral and psychological symptoms of dementia
Cognitive-behavioral therapy	Talking therapy that helps people to understand links between their thoughts, feelings and behaviors, and use this understanding to make positive changes
Recreational activities	Scrapbooking activities, housework and daily tasks, gardening activities
Environmental design	Use of natural light; providing good tonal contrast between flooring, skirtings, walls and doors; minimizing noise sources and ensuring good acoustics by construction and sound absorbent materials, such as floating floors and decorative acoustic wall panels
Cognitive stimulation	Program of themed activities (e.g., discussion of past and present events and topics of interest, word games, puzzles) designed to increase people with dementia’s cognitive and social functioning, and ultimately, their quality of life
Music therapy	Therapy aimed at stimulating different brain areas thus helping the person to express feelings and connect with past memories (e.g., playing music that is significant, listening to favourite pieces of recorded music, singing)
Aromatherapy	Therapy based on the use of aromatic plants or essential oils to reduce symptoms of anxiety and depression. The mechanism consists of the activation of the olfactory receptors and, consequently, of the brain areas associated with emotional regulation
Animal-assisted therapy	Supportive goal-oriented intervention based on human-animal interaction. It allows people with dementia to initiate a social interaction with an animal (dog, horse) in a controlled manner. It is associated with decreased loneliness and agitation, and increased motivation, pleasure and relaxation
Reality orientation	Therapy aimed at increasing cognitive stimulation by orienting people with dementia to the present (e.g., talking about orientation, including the day, time of day, date and season; using people’s name frequently; discussing current events)
Memory training	Program designed to improve people’s attention, concentration, and working and long-term memory (e.g., number mnemonics, story mnemonics, the method of loci)
Validation	Therapy aimed at promoting carer’s ability to listen attentively and respond respectfully to the person with dementia, who can struggle to express basic needs (e.g., use of a clear, low-pitched, and loving tone of voice; eye contact; avoiding to argue)
Emotion-oriented care	Care including different approaches (e.g., validation, reminiscence, sensory integration) designed to increase people with dementia’s emotional and social functioning and, ultimately, their quality of life. Focus is on supporting them in the process of coping with the disease, by linking up with individual functional possibilities, and the person’s subjective experience

Note: Retrieved from [8].

**Table 2:** Overview of National Dementia Plans and Strategies across Europe.

Country	Name and Year of Publication	Source				Categories		
		AE	ADI	Google	Available on Request (*n* = 1)	Treatment	Education	Research
		(*n* = 15)	(*n* = 8)	(*n* = 2)		(*n* = 26)	(*n* = 12)	(*n* = 7)
Austria	National Dementia Strategy: Living well with dementia (2015)		X			X		
Belgium	Flanders Dementia Strategy (2016–2019)	X				X		
Cyprus	National strategic Plan for Dementia (2012–2017)				X	X		
Czech Republic	National Action Plan for Alzheimer’s disease and other related diseases (2016–2019)	X				X	X	
Denmark	A safe and dignity life with dementia: National Dementia Action Plan (2017–2025)		X			X	X	
Finland	National Memory Program: Creating a ‘Memory friendly’ Finland (2013–2020)	X				X	X	X
France	National Plan for neurodegenerative diseases (2014–2019)	X				X	X	X
Germany	National Dementia Strategy (2020)		X			X		X
Greece	National Action Plan for Dementia-Alzheimer’s disease (2015–2020)		X			X		
Iceland	Action Plan for services for people with dementia (2020)	X				X		
Ireland	The Irish National Dementia Strategy (2014)	X				X	X	X
Israel	Addressing Alzheimer´s and other types of dementia: Israeli National Strategy (2013)	X				X		
Italy	Italian National Dementia Strategy (2014)	X				X	X	
Luxembourg	Final report of the Steering Committee on the development of a National Dementia Action Plan (2013)	X				X		
Malta	Empowering change: National Dementia Strategy in the Maltese Islands (2015–2023)	X				X	X	X
Netherlands	Dementia Delta Plan (2012–2020)		X			X	X	X
Norway	Dementia Plan: A more dementia-friendly society (2015)	X				X	X	
Portugal	Action Plan and Budget (2018)			X		X	X	
Slovenia	Dementia Control Strategy within 2020 (2016)	X				X	X	
Spain	Comprehensive Plan for Alzheimer’s and other dementias (2019–2023)		X			X	X	X
Sweden	National Strategy for caring for people with dementia (2018)			X		X		
Switzerland	National Dementia Strategy (2014–2019): Achieved results (2014–2016) and priorities (2017–2019)		X			X		
United Kingdom-England	Living well with dementia: National Dementia Strategy (2009)	X				X		
United Kingdom-Northern Ireland	Improving dementia services in Northern Ireland: A regional Strategy (2011)	X				X		
United Kingdom-Scotland	Scotland’s National Dementia Strategy (2017–2020)		X			X		
United Kingdom-Wales	Dementia Action Plan for Wales (2018–2022)	X				X		

Notes: Alzheimer Europe (AE) members. Retrieved from [33]. Abbreviations: ADI, Alzheimer Disease International. Bosnia Herzegovina, Bulgaria, Croatia, Estonia, Hungaria, Jersey, Montenegro, North Macedonia, Poland, Romania, Slovakia, and Turkey do not have a National Dementia Plan/Strategy.

**Table 3:** Key points and recommendations.

Key Points and Recommendations
There is increasing evidence supporting the use of psychosocial interventions in dementia care. However, concrete actions to translate research into practice are sparse and inconsistent worldwide. Policies serve as the bases for the translation of research findings into everyday clinical practice. The European dementia strategies and plans refer to a model of integrated care and support consisting of medical treatments and psychosocial interventions. However, the amount of information on the latter differs across country policies, with major gaps in the areas of education and research. Policies should be harmonized across Europe and based on a comprehensive and well-integrated system of care, where psychosocial care and interventions are fully embedded. Specifically, they should provide a clearer picture of how psychosocial care and interventions would be enabled and assessed. Only those psychosocial interventions for people with dementia with substantial evidence for efficacy should be recommended. Aims as well as recommendations for their use in different types and stages of dementia should be clearly stated. The same methodology should be followed when addressing the issue of psychosocial interventions for informal carers. Each policy should emphasize the need for developing evidence-based education and training programs. Aims as well as target groups should be identified. To increase public awareness, equal attention should be paid to community education. Each policy should promote the need for research on the effectiveness of psychosocial interventions, with focus on identifying country-specific barriers and facilitators to their implementation. Policy changes may be necessary, but not sufficient for an effective implementation of psychosocial interventions into practice. Multiple and simultaneous actions (e.g., political efforts, adequate financial investments, service design) are needed. All of them should be based on the adoption of the person-centred model of care.

## Data Availability

The data that support the findings of this study are available from the corresponding author, upon reasonable request.

## Supplementary Materials

The following is available online at https://www.mdpi.com/1660-4601/18/7/3422/s1, Table S1: Coding structure with categories, subcategories and codes.
