# Supplementary material for: The Integration of Psychosocial Care into National Dementia Strategies across Europe: Evidence from the Skills in DEmentia Care (SiDECar) Project
Source: Int J Environ Res Public Health. 2021 Mar 25;18(7):3422. doi: 10.3390/ijerph18073422 (PMC8036745; doi:10.3390/ijerph18073422)
Supplement: Supplementary file 1 [file ijerph-18-03422-s001.pdf]

Supplementary Table S1: Coding structure with categories, subcategories and codes.

Categories	Subcategories	Codes
Treatment	Person-centred conceptual framework	o Patient as a person<br>o Identity<br>o Needs assessment<br>o Empowerment<br>o Autonomy<br>o Inclusion in society<br>o Engagement<br>o Meaningful life<br>o Tailored support<br>o Agency<br>o Shared decision making
	Psychosocial interventions	o Psychosocial interventions recommended<br>o Targets of psychosocial interventions<br>o Dementia-friendly environments<br>o Adaptation of physical environment<br>o Assistive technology
	Health and social services networks	o Service partnerships<br>o Provision of various services<br>o Multiprofessional teams<br>o Resource sharing<br>o Integration of services<br>o Easy access to services<br>o Coordination of service delivery
Education		o Education and training programs<br>o Target groups<br>o Evidence-based programs<br>o Flexible programs<br>o Person-centred programs<br>o Program content<br>o Education based on common guidelines<br>o Continuing education<br>o Education for clinical practice<br>o Education for society<br>o ABC education programs
Research		o Need for research<br>o Effectiveness studies<br>o Implementation research<br>o Ecological studies<br>o Research and innovations in healthcare<br>o Research and innovations in technology
